# Supplementary material for: Maximizing and evaluating the impact of test-trace-isolate programs: A modeling study
Source: PLoS Med. 2021 Apr 30;18(4):e1003585. doi: 10.1371/journal.pmed.1003585 (PMC8121292; doi:10.1371/journal.pmed.1003585)
Supplement: S1 Text — Model details, input parameters, and sensitivity analyses. (PDF) [file pmed.1003585.s001.pdf]

S1 Text. Supplementary Information
Maximizing and evaluating the impact of test-trace-isolate programs: a modeling study

Kyra H. Grantz⋆, Elizabeth C. Lee⋆, Lucy D'Agostino McGowan, Kyu Han Lee, C. Jessica E. Metcalf, Emily S. Gurley, Justin Lessler
⋆*denotes equal contribution*

Supplementary Methods

Mathematical Model

Infection Compartments

In this model, we assume that disease transmission occurs in discrete generations and that all infections may be classified into compartments, which are defined as elements of a 1×9 surveillance-quarantine-community (DQC) matrix:

$$DQC = \begin{bmatrix} D(.,s,.) & D(.,a,.) & Q(Ds,c,.) & Q(Ds,h,.) & Q(Da,c,.) & Q(Da,h,.) & Q(Q,.,.) & C(.,s,.) & C(.,a,.) \end{bmatrix} \quad (7)$$

where the broad D, Q, and C classes describe infections identified through surveillance, infections that were quarantined due to contact tracing efforts, and infections that remained undetected in the community. The DQC classes are further differentiated by characteristics of their infector (x), ego characteristics (y), and characteristics of their infectees (z) in a three element tuple (x,y,z). We use this standard tuple notation across infection compartments and parameters for ease of understanding. Any element of the tuple filled with . means that that type of characteristic is not applicable. The DQC compartments are defined in Table A.

Table A: **Detected and Isolated - Quarantined - Community (DQC) infection compartments**

Compartment	Description
$D(.,s,.)$	symptomatic infections detected through surveillance
$D(.,a,.)$	asymptomatic infections detected through surveillance
$Q(Ds,c,.)$	infected community contacts of a surveillance-detected symptomatic infection that are in quarantine
$Q(Ds,h,.)$	infected household contacts of a surveillance-detected symptomatic infection that are in quarantine
$Q(Da,c,.)$	infected community contacts of a surveillance-detected asymptomatic infection that are in quarantine
$Q(Da,h,.)$	infected household contacts of a surveillance-detected asymptomatic infection that are in quarantine
$Q(Q,.,.)$	infected (household or community) contacts of a quarantined infection that are in quarantine
$C(.,s,.)$	symptomatic infections that remain undetected in the community
$C(.,a,.)$	asymptomatic infections that remain undetected in the community

The elements of the DQC matrix refer to the proportion of total infections in each compartment for a given disease generation t, and the sum of any individual DQC matrix is 1.

Recursive propagation of infections

We can propagate infections across disease generations recursively:

$$DQC_{t+1} = (DQC_t)(INFECT)(DETECT), \tag{8}$$

where $INFECT$ is a 9×6 matrix describing the rates of transition from one disease generation to the next, and DETECT is a 6×9 matrix describing the probability that infections in the next generation are identified by surveillance, quarantined, or undetected in the community in the DQC matrix for generation $t + 1$.

$INFECT$ is a sparse matrix of transition rates from DQC compartments to infections caused by specific DQC compartments. While not strictly necessary, for ease of accounting, we notate the number of next-generation infections that are derived from each DQC compartment in Table B. We specify only six next-generation infection states because we group all infections caused by quarantined individuals into a single $I(Q, ., .)$ class; this means that all infections derived from quarantined individuals have the same probability of assignment to the appropriate compartments in the DQC_{t+1} matrix, regardless of who infected them and whether they were community or household contacts of those index infections.

Table B: **Infections in the next generation, by infector**

**Infection Type**	**Description**
$I(Ds, c, .)$	community contacts infected by surveillance-detected symptomatic individuals
$I(Ds, h, .)$	household contacts infected by surveillance-detected symptomatic individuals
$I(Da, c, .)$	community contacts infected by surveillance-detected asymptomatic individuals
$I(Da, h, .)$	household contacts infected by surveillance-detected asymptomatic individuals
$I(Q, ., .)$	(community or household) contacts infected by quarantined individuals
$I(C, ., .)$	infected (household or community) contacts of a quarantined infection that are in quarantine

The elements of the $INFECT$ matrix represent the transmission rates between DQC compartments and the infections in the next generation ($I(x, y, z)$), described by the notation *compartment* $\rightarrow$ *infection state*:

$INFECT =$

$$\begin{bmatrix} D(.,s,.) \rightarrow I(Ds,c,.) & D(.,s,.) \rightarrow I(Ds,h,.) & 0 & 0 & 0 & 0 \\ 0 & 0 & D(.,a,.) \rightarrow I(Da,c,.) & D(.,a,.) \rightarrow I(Da,h,.) & 0 & 0 \\ 0 & 0 & 0 & 0 & Q(Ds,c,.) \rightarrow I(Q,.,.) & 0 \\ 0 & 0 & 0 & 0 & Q(Ds,h,.) \rightarrow I(Q,.,.) & 0 \\ 0 & 0 & 0 & 0 & Q(Da,c,.) \rightarrow I(Q,.,.) & 0 \\ 0 & 0 & 0 & 0 & Q(Da,h,.) \rightarrow I(Q,.,.) & 0 \\ 0 & 0 & 0 & 0 & Q_Q \rightarrow I(Q,.,.) & 0 \\ 0 & 0 & 0 & 0 & 0 & C(.,s,.) \rightarrow I(C,.,.) \\ 0 & 0 & 0 & 0 & 0 & C(.,a,.) \rightarrow I(C,.,.) \end{bmatrix} \tag{9}$$

$DETECT$ is a matrix of transition probabilities from infection states to DQC compartments in the next generation.

$DETECT =$

$$\begin{bmatrix} I(Ds,c,.) \rightarrow D(.,s,.) & I(Ds,c,.) \rightarrow D(.,a,.) & I(Ds,c,.) \rightarrow Q(Ds,c,.) & 0 & 0 & 0 & 0 & I(Ds,c,.) \rightarrow C(.,s,.) & I(Ds,c,.) \rightarrow C(.,a,.) \\ I(Ds,h,.) \rightarrow D(.,s,.) & I(Ds,h,.) \rightarrow D(.,a,.) & 0 & I(Ds,h,.) \rightarrow Q(Ds,h,.) & 0 & 0 & 0 & I(Ds,h,.) \rightarrow C(.,s,.) & I(Ds,h,.) \rightarrow C(.,a,.) \\ I(Da,c,.) \rightarrow D(.,s,.) & I(Da,c,.) \rightarrow D(.,a,.) & 0 & 0 & I(Da,c,.) \rightarrow Q(Da,c,.) & 0 & 0 & I(Da,c,.) \rightarrow C(.,s,.) & I(Da,c,.) \rightarrow C(.,a,.) \\ I(Da,h,.) \rightarrow D(.,s,.) & I(Da,h,.) \rightarrow D(.,a,.) & 0 & 0 & 0 & I(Da,h,.) \rightarrow Q(Da,h,.) & 0 & I(Da,h,.) \rightarrow C(.,s,.) & I(Da,h,.) \rightarrow C(.,a,.) \\ I(Q,.,.) \rightarrow D(.,s,.) & I(Q,.,.) \rightarrow D(.,a,.) & 0 & 0 & 0 & 0 & I(Q,.,.) \rightarrow Q(Q,.,.) & I(Q,.,.) \rightarrow C(.,s,.) & I(Q,.,.) \rightarrow C(.,a,.) \\ I(C,.,.) \rightarrow D(.,s,.) & I(C,.,.) \rightarrow D(.,a,.) & 0 & 0 & 0 & 0 & 0 & I(C,.,.) \rightarrow C(.,s,.) & I(C,.,.) \rightarrow C(.,a,.) \end{bmatrix} \tag{10}$$

Transmission may differ based on the characteristics of the infecting individual (symptomatic individuals may shed more than asymptomatic ones) and the type of infectee contact (household contacts may have greater relative risk of infection than community contacts). Consequently, the transition probabilities described by the $INFECT$ matrix may include different variations of the reproductive number R. Using the same tuple notation described above, we describe $R(x, y, z)$, where R represents the population-wide baseline reproductive number. Note that $R(., ., c)$ and $R(., ., h)$ are shown here only for demonstrative purposes and they are not used by themselves. Parameters are defined in Table C.

$$R(., s, .) = R/(\alpha\kappa - \alpha + 1) \tag{11}$$

$$R(., a, .) = \kappa R/(\alpha\kappa - \alpha + 1) \tag{12}$$

$$R(., ., c) = R/(\eta\nu - \eta + 1) \tag{13}$$

$$R(., ., h) = \nu R/(\eta\nu - \eta + 1) \tag{14}$$

$$R(., a, h) = \kappa\nu R/(\alpha\kappa - \alpha + 1)(\eta\nu - \eta + 1) \tag{15}$$

$$R(., a, c) = \kappa R/(\alpha\kappa - \alpha + 1)(\eta\nu - \eta + 1) \tag{16}$$

$$R(., s, h) = \nu R/(\alpha\kappa - \alpha + 1)(\eta\nu - \eta + 1) \tag{17}$$

$$R(., s, c) = R/(\alpha\kappa - \alpha + 1)(\eta\nu - \eta + 1) \tag{18}$$

We define the truncation in infectiousness due to isolation of an index case ($\gamma_{D(y)}$), and therefore truncation of the infection period as:

$$\gamma_{D(y)} = \int_{-\infty}^{\tau_{D(y)}} f(x)dx \tag{19}$$

where $f(x)$ is the distribution of infectiousness, which is a function of x days since symptom onset. The integral from $-\infty$ to $\tau_{D(y)}$ represents the proportion of total infectiousness where a transmission event may occur before the effective isolation of an index case of type y.

We derived the distribution of infectiousness (Gamma: shape = 21.13, rate = 1.59, offset= -12.27) relative to symptom onset from a previously published work [42], which had different estimates of the generation time (5.8 days to our baseline 6.5 days) and incubation period (5.2 days to our baseline 5.5 days) (Table C). We aligned their estimate for the infectiousness distribution to our generation time and incubation period assumptions, by holding the rate parameter constant and solving for the shape parameter for $f(x)$ in the equation that follows (Table C):

$$\text{generation time} = \mathbb{E}[X] + \text{incubation period} \tag{20}$$

where X is the gamma-distributed random variable representing time from primary symptom onset to secondary infection.

We used these same parameters to develop a distribution of infectiousness of secondary cases of type y, $g(x)$, as a function of the time from their infector's time of symptom onset to contact quarantine, $\tau_{Q(y)}$. Once again assuming a gamma distribution, we hold the rate equal to 1.59 and offset equal to -12.27 for both $f(x)$ and $g(x)$ and solve for the shape of $g(x)$ using the

incubation period and shape, rate, and offset of $f(x)$.

We thus define the reduction in infectiousness due to quarantine (and subsequent isolation) of infected contacts as:

$$\gamma_{Q(y)} = \int_{-\infty}^{\tau_{Q(y)}} g(x)dx \tag{21}$$

In both cases, it is assumed that case isolation is perfectly effective (that is, all transmission is stopped once a case is isolated). This assumption can be relaxed by reducing the proportion of cases assumed to be isolated or quarantined by the assumed reductions in isolation effectiveness (that is, isolating 50% of cases at 100% effectiveness is equivalent to isolating 100% of cases at 50% effectiveness).

The equations governing the $INFECT$ matrix are then as follows:

$$D(.,s,.) \to I(Ds,c,.) = (1-\eta)\gamma_{D(.,s,.)}R(.,s,c) \tag{22}$$

$$D(.,s,.) \to I(Ds,h,.) = \eta\gamma_{D(.,s,.)}R(.,s,h) \tag{23}$$

$$D(.,a,.) \to I(Da,c,.) = (1-\eta)\gamma_{D(.,a,.)}R(.,a,c) \tag{24}$$

$$D(.,a,.) \to I(Da,h,.) = \eta\gamma_{D(.,a,.)}R(.,a,h) \tag{25}$$

$$Q(Ds,c,.) \to I(Q,.,.) = \gamma_{Q(Ds,c,.)}R \tag{26}$$

$$Q(Ds,h,.) \to I(Q,.,.) = \gamma_{Q(Ds,h,.)}R \tag{27}$$

$$Q(Da,c,.) \to I(Q,.,.) = \gamma_{Q(Da,c,.)}R \tag{28}$$

$$Q(Da,h,.) \to I(Q,.,.) = \gamma_{Q(Da,h,.)}R \tag{29}$$

$$Q(Q,.,.) \to I(Q,.,.) = \gamma_{Q(Q,.,.)}R \tag{30}$$

$$C(.,s,.) \to I(C,.,.) = R(.,s,.) \tag{31}$$

$$C(.,a,.) \to I(C,.,.) = R(.,a,.) \tag{32}$$

The $DETECT$ matrix assigns infections to DQC compartments in the next generation. In the baseline model, we assume that quarantine is perfectly effective, such that any infected individual placed under quarantine will be identified as a case and effectively isolated. For programs which may have reduced the length of quarantine from time of exposure, t_q, a scalar β is applied to each ω term:

$$\beta = 1 - \int_{0}^{t_q} h(x)dx \tag{33}$$

where $h(x)$ is a log-normal distribution of the incubation period [43], such that the proportion of individuals who would have symptom onset greater than the length of quarantine are assumed to be undetected. This is just one way to represent the case detection process among quarantined individuals, which could easily be modified in our framework to reflect different detection schema.

The equations governing the $DETECT$ matrix are as follows:

$$I(Ds, c, .) \rightarrow D(., s, .) = (1-\alpha)(1-\beta\omega(., c, .))\rho_s \tag{34}$$

$$I(Ds, c, .) \rightarrow D(., a, .) = \alpha(1-\beta * \omega(., c, .))\rho_a \tag{35}$$

$$I(Ds, c, .) \rightarrow Q(Ds, c, .) = \beta * \omega(., c, .) \tag{36}$$

$$I(Ds, c, .) \rightarrow C(., s, .) = (1-\alpha)(1-\beta * \omega(., c, .))(1-\rho_s) \tag{37}$$

$$I(Ds, c, .) \rightarrow C(., a, .) = \alpha(1-\beta * \omega(., c, .))(1-\rho_a) \tag{38}$$

$$I(Ds, h, .) \rightarrow D(., s, .) = (1-\alpha)(1-\beta * \omega(., h, .))\rho_s \tag{39}$$

$$I(Ds, h, .) \rightarrow D(., a, .) = \alpha(1-\beta * \omega(., h, .))\rho_a \tag{40}$$

$$I(Ds, h, .) \rightarrow Q(Ds, h, .) = \beta * \omega(., h, .) \tag{41}$$

$$I(Ds, h, .) \rightarrow C(., s, .) = (1-\alpha)(1-\beta * \omega(., h, .))(1-\rho_s) \tag{42}$$

$$I(Ds, h, .) \rightarrow C(., a, .) = \alpha(1-\beta * \omega(., h, .))(1-\rho_a) \tag{43}$$

$$I(Da, c, .) \rightarrow D(., s, .) = (1-\alpha)(1-\beta * \omega(., c, .))\rho_s \tag{44}$$

$$I(Da, c, .) \rightarrow D(., a, .) = \alpha(1-\beta * \omega(., c, .))\rho_a \tag{45}$$

$$I(Da, c, .) \rightarrow Q(Da, c, .) = \beta * \omega(., c, .) \tag{46}$$

$$I(Da, c, .) \rightarrow C(., s, .) = (1-\alpha)(1-\beta * \omega(., c, .))(1-\rho_s \tag{47}$$

$$I(Da, c, .) \rightarrow C(., a, .) = \alpha(1-\beta * \omega(., c, .))(1-\rho_a) \tag{48}$$

$$I(Da, h, .) \rightarrow D(., s, .) = (1-\alpha)(1-\beta * \omega(., h, .))\rho_s \tag{49}$$

$$I(Da, h, .) \rightarrow D(., a, .) = \alpha(1-\beta * \omega(., h, .))\rho_a \tag{50}$$

$$I(Da, h, .) \rightarrow Q(Da, h, .) = \beta * \omega(., h, .) \tag{51}$$

$$I(Da, h, .) \rightarrow C(., s, .) = (1-\alpha)(1-\beta * \omega(., h, .))(1-\rho_s) \tag{52}$$

$$I(Da, h, .) \rightarrow C(., a, .) = \alpha(1-\beta * \omega(., h, .))(1-\rho_a) \tag{53}$$

$$I(Q, ., .) \rightarrow D(., s, .) = (1-\alpha)(1-\beta * \omega(Q, ., .))\rho_s \tag{54}$$

$$I(Q, ., .) \rightarrow D(., a, .) = \alpha(1-\beta * \omega(Q, ., .))\rho_a \tag{55}$$

$$I(Q, ., .) \rightarrow Q(Q, ., .) = \beta * \omega(Q, ., .) \tag{56}$$

$$I(Q, ., .) \rightarrow C(., s, .) = (1-\alpha)(1-\beta * \omega(Q, ., .))(1-\rho_s) \tag{57}$$

$$I(Q, ., .) \rightarrow C(., a, .) = \alpha(1-\beta * \omega(Q, ., .))(1-\rho_a) \tag{58}$$

$$I(C, ., .) \rightarrow D(., s, .) = (1-\alpha)\rho_s \tag{59}$$

$$I(C, ., .) \rightarrow D(., a, .) = \alpha\rho_a \tag{60}$$

$$I(C, ., .) \rightarrow C(., s, .) = (1-\alpha)(1-\rho_s) \tag{61}$$

$$I(C, ., .) \rightarrow C(., a, .) = \alpha(1-\rho_a) \tag{62}$$

Table C: **Model parameters and default values** of fixed disease transmission and natural history values.

Parameter	Description	Default	Ref.
**Natural history**			
α	proportion of infections that are asymptomatic	0	baseline assumption; plausible values [18]
-	Mean generation time (time between infection of index case and secondary infection)	6.5 days	[15]
-	Mean incubation period (time between infection and symptom onset of index case)	5.5 days	[43, 44]
-	Shape, rate, and offset of gamma distribution of infectiousness relative to time from symptom onset, $f(x)$	21.13, 1.59, -12.27	derived from [42]
**Disease transmission**			
N	number of infections (*stochastic model only*)	-	
R	baseline reproductive number for the population	2.5	
$R(., y, z)$	reproductive number for y-type index cases transmitting to z-type infectees	-	
η	proportion of contacts that are household contacts	1	baseline assumption; plausible values [45]
ν	relative risk of infection among household contacts compared to community contacts	4	similar to [37]
κ	relative transmissibility for asymptomatic relative to symptomatic infected individuals	0	baseline assumption
$\gamma_{D(y)}, \gamma_{Q(y)}$	infectiousness multiplier, accounting for the reduction in y type individual's infectious period due to isolation (γ_D) or quarantine (γ_Q)	-	
$\tau_{D(y)}$	time delay from y type individual's symptom onset to isolation	-	
$\tau_{Q(y)}$	time delay from infector's symptom onset to quarantine of y type contact	-	
θ	Overdispersion parameter (*stochastic model only*)	0.1	[19]
**Disease detection**			
ρ_a	proportion of asymptomatic infections that are detected by surveillance	-	
ρ_s	proportion of symptomatic infections that are detected by surveillance	-	
$\omega(x, y, .)$	proportion of x-caused infections or y type individual's contacts that are identified and quarantined	-	
β	proportion of quarantined, infected contacts which are identified as cases and isolated	1	

Parameters marked with '-' have no default value because they vary across the multiple scenarios presented. Unless otherwise stated, all scenarios assume that $\rho_a = \rho_s$, though this assumption has no effect when $\kappa = 0$, and that $\omega(., h.) = \omega(., c, .)$, though this assumption has no effect when $\eta = 1$.

Supplementary Figures

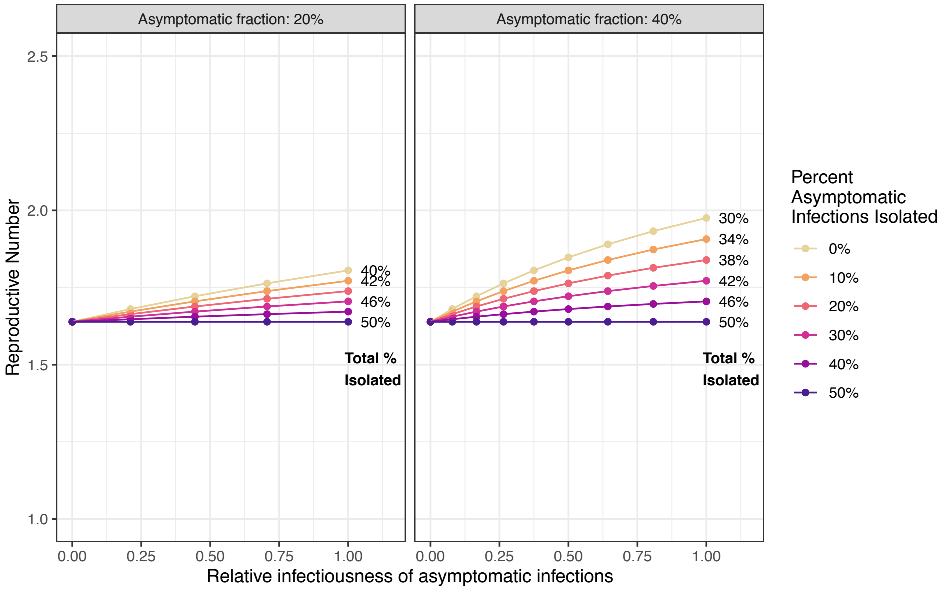

Fig A: Additional benefits from isolation of asymptomatic, infected individuals, for a scenario with high case isolation completeness among symptomatic infections (50%) and high contact quarantine completeness (70%) on average 4 days after case symptom onset. Improving asymptomatic case isolation completeness will have a larger impact when the the relative infectiousness of asymptomatic infections, compared to symptomatic infections, approaches 1 (*x-axis*) and when the fraction of asymptomatic infections in the population is higher (*asymptomatic fraction*). Numbers by each line show the percent of all infections (symptomatic and asymptomatic) that are isolated.

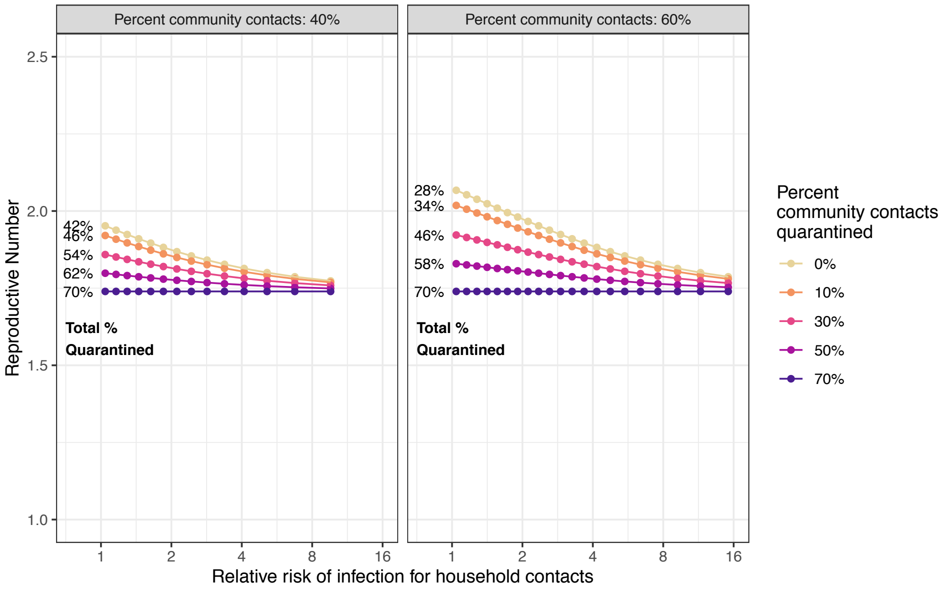

Fig B: Additional benefits from quarantine of community (non-household) contacts, for a scenario with high case isolation completeness (50%) and high household contact quarantine completeness (70%) on average 4 days after case symptom onset. Improving community contact quarantine completeness will have limited impact when the the relative risk of infection among household infections, compared to community infections, is high (*x-axis*) and when the percent of contacts occurring outside of the household is lower. Numbers by each line show the percent of all contacts (household and community) that are quarantined.

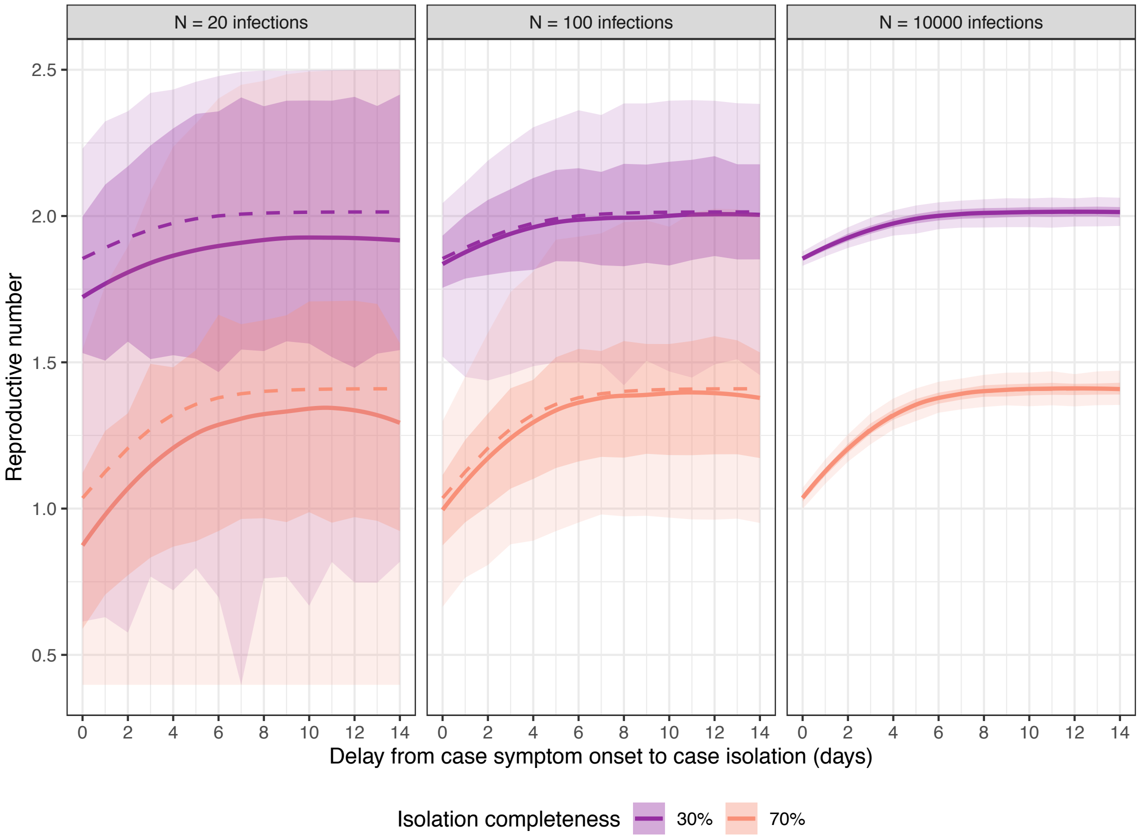

Fig C: Impacts of overdispersion and stochasticity on model estimates of the reproductive number. Solid lines show the mean reproductive number across 1000 simulations (darker shaded regions, interquartile range; lighter shaded regions, 95% confidence interval) for two scenarios with highly-effective contact tracing (70% quarantine completeness on average 4 days after case symptom onset), with either 20, 100, or 10000 total infections, and with overdispersion parameter $\theta = 0.1$. Dashed lines show equivalent results without overdispersion.

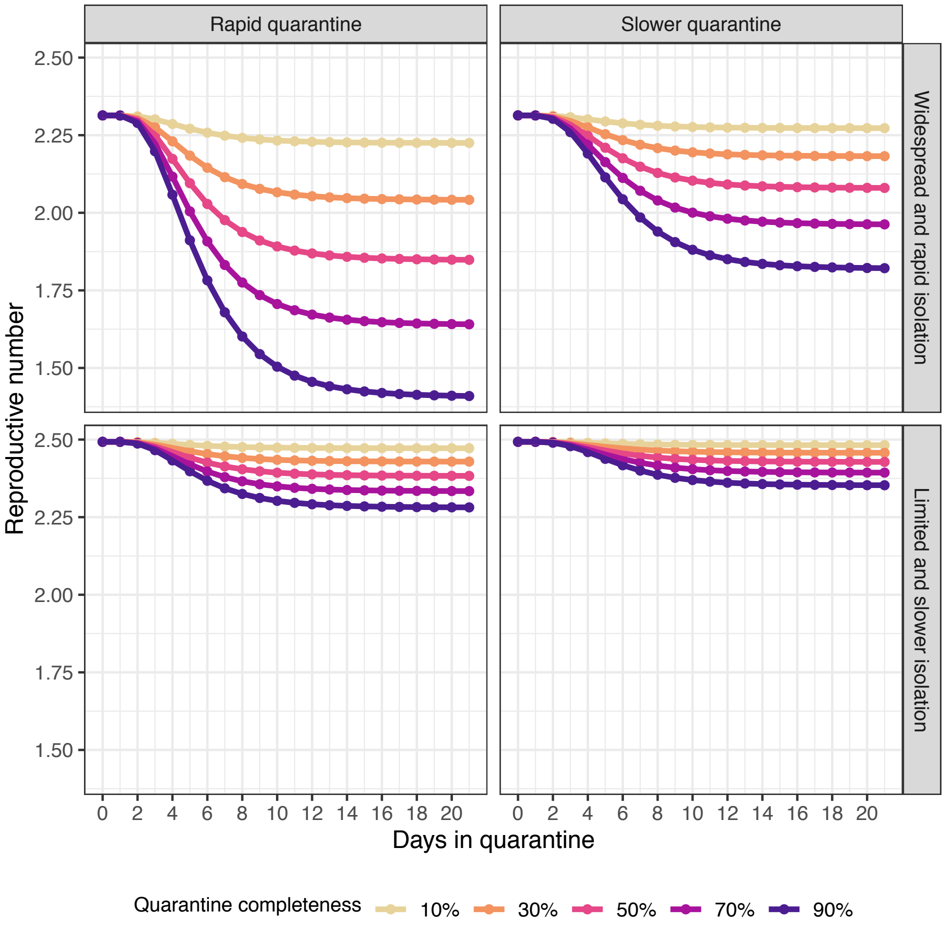

Fig D: Impact of quarantine duration on model estimates of the reproductive number. Four scenarios are depicted with combinations of rapid quarantine (on average 4 days after case symptom onset) or slower quarantine (on average 8 days after case symptom onset) and widespread and rapid isolation (50% isolated on average 4 days after case symptom onset) or limited and slower isolation (10% isolated on average 7 days after case symptom onset) Any infected contact with symptom onset greater than the average duration in quarantine is assumed to be undetected.

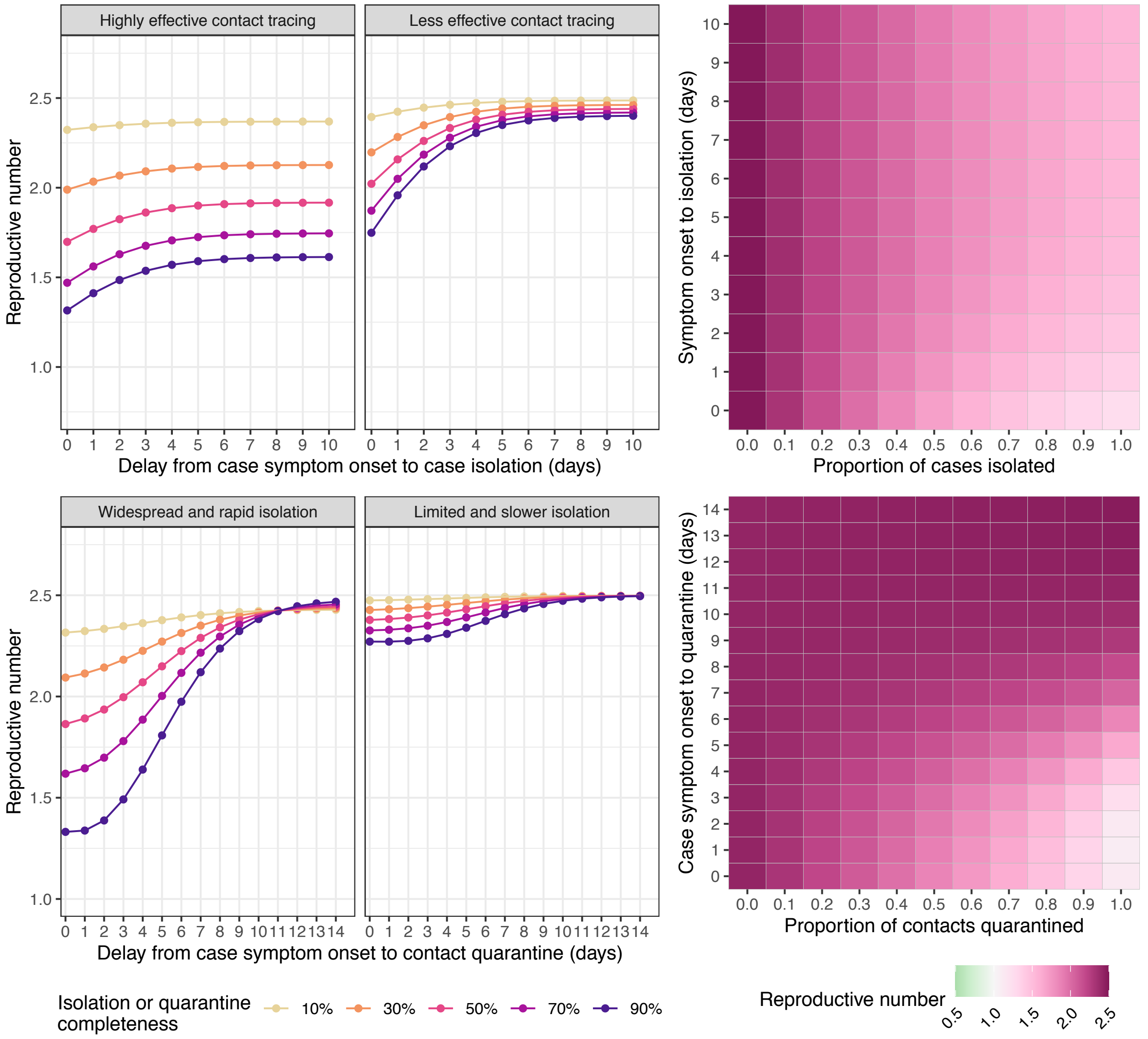

Fig E: Improvements to case isolation and contact quarantine where the generation time is 5 days: A) Impact of case isolation timing (x-axis) and completeness (line colors) on the effective reproductive number (y-axis) for a highly effective contact tracing program (left) and a less effective contact tracing program (center). Heat map (right) of the effective reproductive number across a range of case isolation timing (y-axis) and completeness (x-axis) scenarios, assuming that contact tracing is highly effective. B) Impact of contact tracing timing (x-axis) and completeness (line colors) on the effective reproductive number (y-axis) for a widespread and rapid case isolation scenario (left) and a less effective and slower case isolation scenario (center). Heat map (right) of the effective reproductive number across a range of contact tracing timing (y-axis) and completeness (x-axis) scenarios, assuming that detection and isolation of index cases is widespread and rapid.

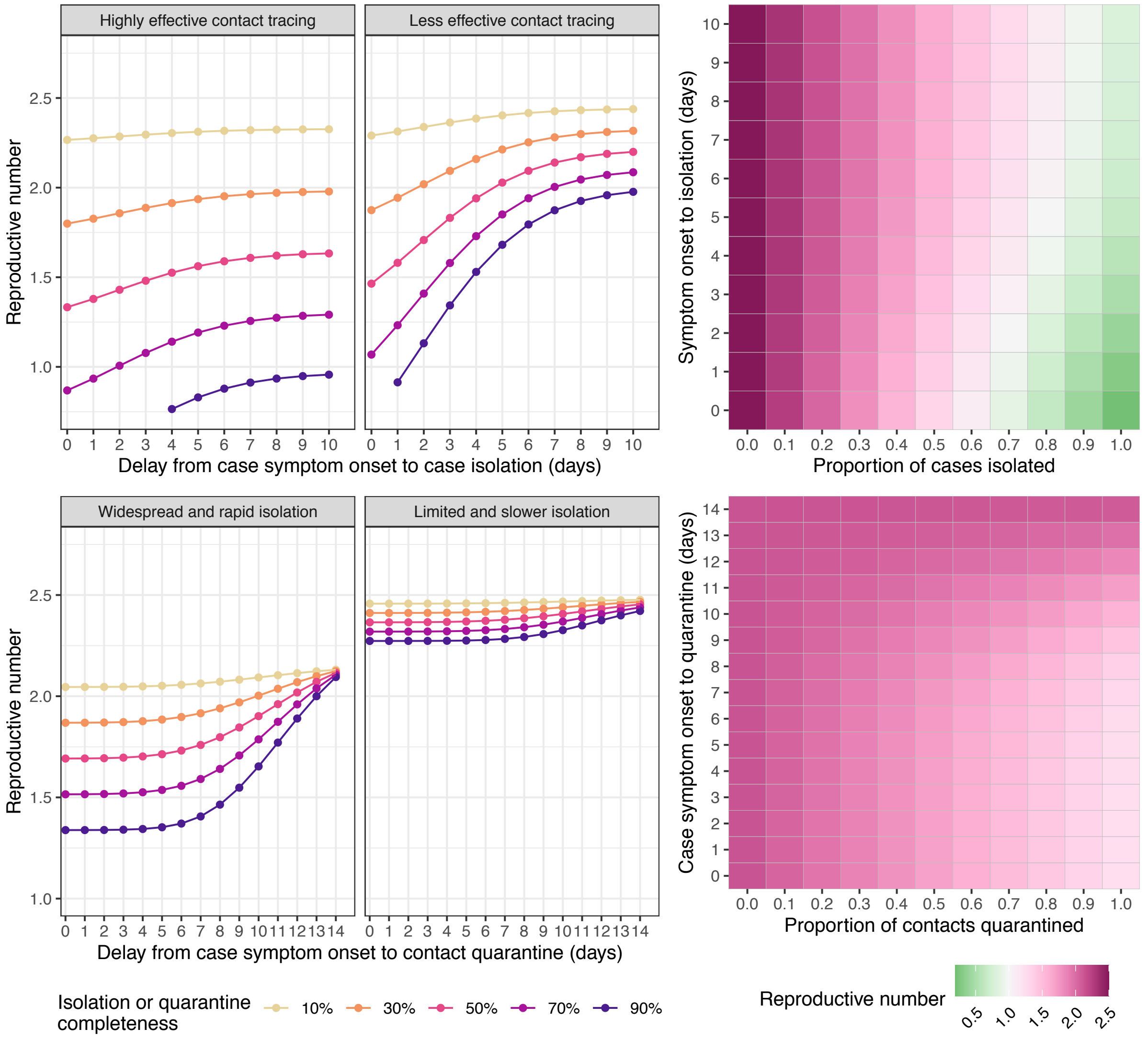

Fig F: Improvements to case isolation and contact quarantine where the generation time is 8 days: A) Impact of case isolation timing (x-axis) and completeness (line colors) on the effective reproductive number (y-axis) for a highly effective contact tracing program (left) and a less effective contact tracing program (center). Heat map (right) of the effective reproductive number across a range of case isolation timing (y-axis) and completeness (x-axis) scenarios, assuming that contact tracing is highly effective. B) Impact of contact tracing timing (x-axis) and completeness (line colors) on the effective reproductive number (y-axis) for a widespread and rapid case isolation scenario (left) and a less effective and slower case isolation scenario (center). Heat map (right) of the effective reproductive number across a range of contact tracing timing (y-axis) and completeness (x-axis) scenarios, assuming that detection and isolation of index cases is widespread and rapid.

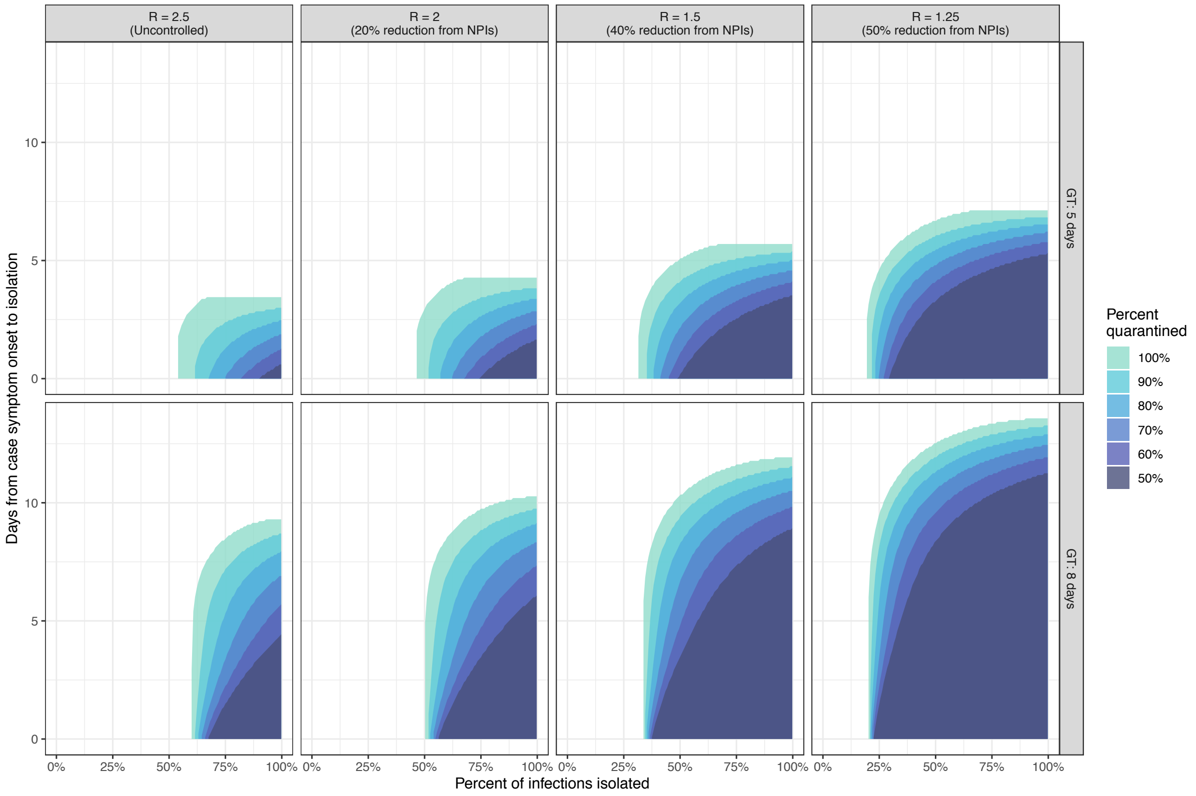

Fig G: Isolation strategies (timing and completeness) capable of achieving $R < 1$ when a given proportion of contacts (50 - 100%) are quarantined on the same day as case isolation. These strategies are shown for two assumptions of the generation time (5 days or 8 days) and for four possible baseline values of R, assuming that other non-pharmaceutical interventions (NPIs) are in effect to reduce transmission from the uncontrolled scenario, $R = 2.5$.

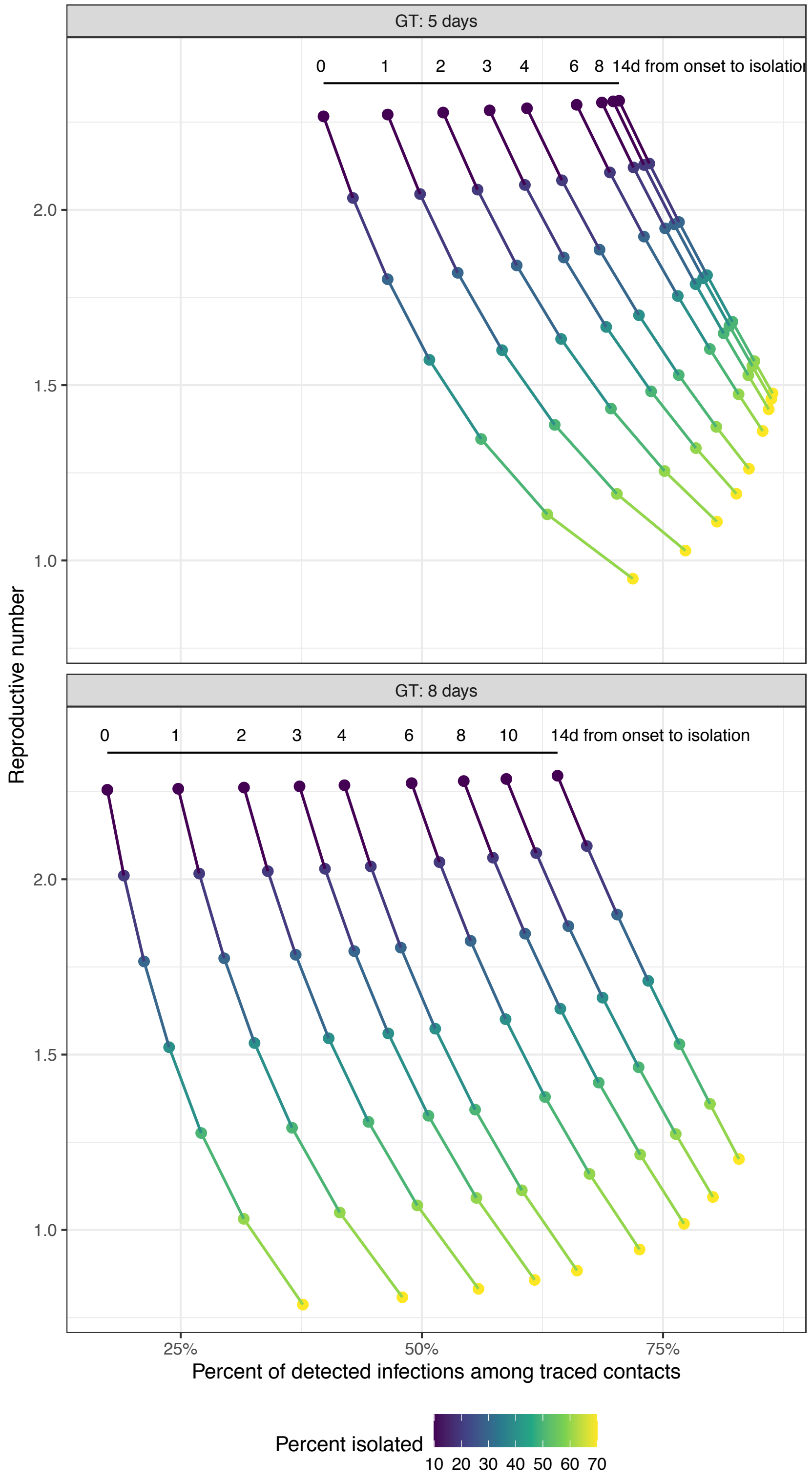

Fig H: Relationship between R and the proportion of detected infections among identified contacts, under two assumptions of the generation time (5 days and 8 days). Each position along a line shows a single test-trace-isolate strategy, with a fixed delay from case symptom onset to isolation (shown in the numbers at the top). Points are colored by the proportion of all infections that are isolated through surveillance or testing.

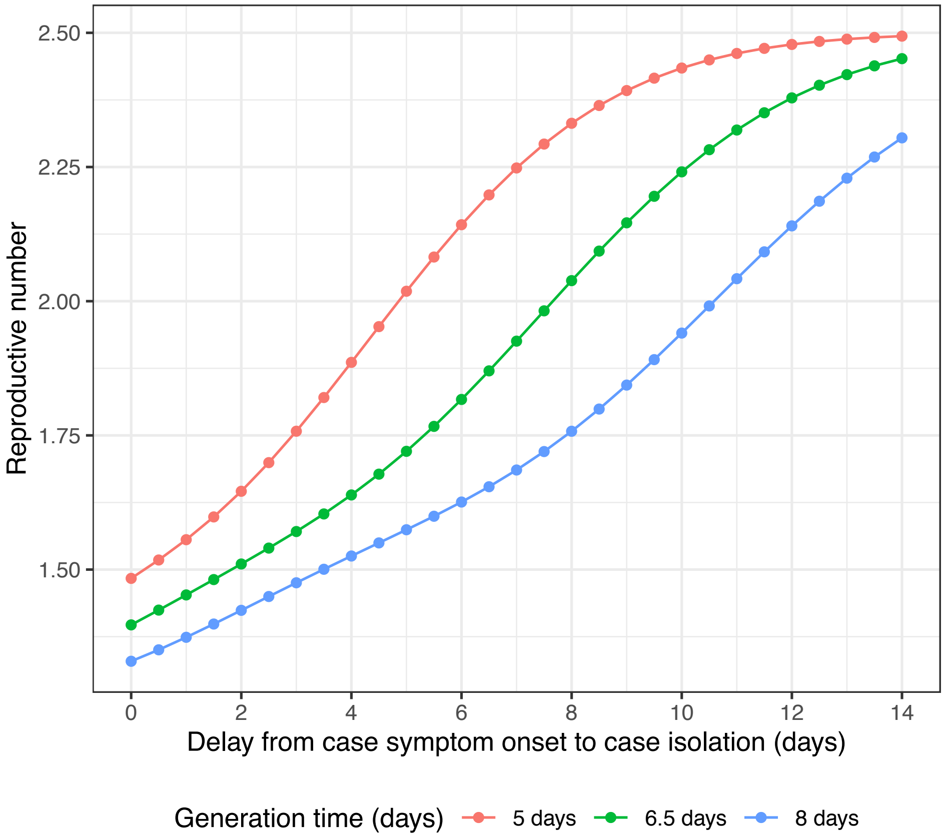

Fig I: Impact of generation time assumption on reproductive number, for a scenario with high case isolation completeness (50%) and high contact quarantine completeness (70%) on the same day as case isolation. A shorter generation time implies that a greater proportion of transmission occurs before or immediately after symptom onset and, hence, that the delay from case symptom onset to case isolation must be shorter to achieve equivalent reductions to the reproductive number.
