# Supplementary material for: Maximizing and evaluating the impact of test-trace-isolate programs: A modeling study
Source: PLoS Med. 2021 Apr 30;18(4):e1003585. doi: 10.1371/journal.pmed.1003585 (PMC8121292; doi:10.1371/journal.pmed.1003585)
Supplement: Attachment — Submitted filename: PMEDICINE-D-20-04296R1_response.pdf [file pmed.1003585.s002.pdf]

JOHNS HOPKINS
BLOOMBERG SCHOOL
of PUBLIC HEALTH

January 18, 2021

Dear Editor:

Thank you for sending us feedback on our manuscript “Maximizing and evaluating the impact of test-trace-isolate programs: a modeling study” (PMEDICINE-D-20-04296R1). We made a number of changes to our manuscript and accompanying tools in response to reviewer comments; most notably, we have extended the sections of our text that describe how isolation and quarantine compliance may be modeled in our framework and added a new model feature which enables examination of the impact of quarantine duration.

Detailed responses to each comment are included below. Please let me know if you have any questions.

Sincerely,

Justin Lessler
Department of Epidemiology
Johns Hopkins Bloomberg School of Public Health
justin@jhu.edu

Requests from the editors:

**In line with the usual journal style and to support information retrieval in search indexes etc, we'd suggest including some designation of the study design/methodological approach in the article title (normally this is in a subtitle after a colon, and here we'd imagine something along the lines of "....: modelling study" might be appropriate).*

We have changed the title to: “Maximizing and evaluating the impact of test-trace-isolate programs: a modeling study”

**PLOS Medicine style for the abstract normally involves including a summary/note about any key limitations of the study methods in the abstract (normally in the last sentence of the Abstract Methods and Findings section). We noted that the reviewers raise queries about the extent to which compliance or adherence with isolation measures are factored into the model - so perhaps this could be mentioned. (see also below for the main discussion section).*
Because the paper addresses a topic of extreme current importance to public health, and the findings could directly influence policy, we'd imagine it may be (if accepted and published) widely read. Therefore it would be good to make sure the abstract, particularly, is framed in a way that can be more clearly understood by nonspecialists and perhaps even lay readers. Some sentences currently are a bit cryptic for non-specialists - eg the following, and we'd suggest reframing this (and some others) so the meaning is more clear: "Formally framing the dynamical process** also indicates that metrics used to evaluate performance of test-trace-isolate, such as the proportion of identified infections among traced contacts, may be misleading". (The part in asterisks is the bit that many readers may stumble over).*

We have rewritten the abstract to read:

“We present a mathematical modeling framework to evaluate the expected reductions in the reproductive number, R, from test-trace-isolate programs. This framework is implemented in a publicly available R package and an online application. We evaluated the effects of completeness in case detection and contact tracing and speed of isolation and quarantine using parameters consistent with COVID-19 transmission (R_0: 2.5, generation time: 6.5 days). We show that R is most sensitive to changes in the proportion of cases detected in almost all scenarios, and other metrics have a reduced impact when case detection levels are low (<30%). Although test-trace-isolate programs can contribute substantially to reducing R, exceptional performance across all metrics is needed to bring R below one through test-trace-isolate alone, highlighting the need for comprehensive control strategies. Results from this model also indicate that metrics used to evaluate performance of test-trace-isolate, such as the proportion of identified infections among traced contacts, may be misleading. While estimates of the impact of test-trace-isolate are sensitive to assumptions about COVID-19 natural history and adherence to isolation and quarantine, our qualitative findings are robust across numerous sensitivity analyses.”

**At this stage, we ask that you include a short, non-technical Author Summary of your research to make findings accessible to a wide audience that includes both scientists and non-scientists. The Author Summary should immediately follow the Abstract in your revised manuscript. This text is subject to editorial change and should be distinct from the scientific abstract. Please see our author guidelines for more information:*
https://nam02.safelinks.protection.outlook.com/?url=https%3A%2F%2Fjournals.plos.org%2Fplosmedicine%2Fs%2Frevising-your-manuscript%23loc-author-summary&data=04%7C01%7Celizabeth.c.lee%40jhu.edu%7Cbc1066e2cec4457c5c0f08d89de2eb04%7C9fa4f438b1e6473b803f86f8aedf0dec%7C0%7C0%7C637432945220883897%7CUnknown%7CTWFpbGZsb3d8eyJWIjoiMC4wLjAwMDAiLCJQIjoiV2luMzIiLCJBTiI6Ik1haWwiLCJXVCI6Mn0%3D%7C1000&sdata=9KLS1Zos27qIdRLGQIBR1UkDfksQXoJJ1Il9WCw46lw%3D&reserved=0

We have added the following Author Summary:

Why Was This Study Done?

- Control measures for the ongoing COVID-19 pandemic rely largely on untargeted interventions, like social distancing, which have high economic and social costs.
- Test-trace-isolate programs, in which known cases are asked to isolate and their contacts are traced and then asked to quarantine, are an attractive option to control the spread of COVID-19 in a more targeted fashion.
- Estimating the impact of test-trace-isolate programs is not straightforward, due to feedback loops between control measures and disease transmission.

What Did the Researchers Do and Find?

- We developed a mathematical modeling framework to assess the potential impact of test-trace-isolate programs.
- In most cases, increasing the percentage of cases successfully isolated will yield the largest relative reductions in disease transmission (as compared to improvements in successful contact quarantine or reductions in time to isolation or quarantine).
- Programs already achieving a high percentage of case isolation will see more substantial gains from improving the speed of case isolation and improving the completeness and speed of contact tracing and quarantine.

What Do These Findings Mean?

- Test-trace-isolate programs must be supported by widespread and expeditious testing and case detection to suppress SARS-CoV-2 transmission.
- Even imperfect test-trace-isolate programs can meaningfully complement other interventions as part of a comprehensive public health response with fewer social and economic costs.
- The modeling framework is publicly available in an interactive web application, and additional teaching materials are available in a free online course.

**Please clarify in the paper if the analytical approach followed here was set out prospectively - please state this (either way) early in the Methods section.*

a) If a prospective analysis plan (from your funding proposal, IRB or other ethics committee submission, study protocol, or other planning document written before analyzing the data) was used in designing the study, please include the relevant prospectively written document with your revised manuscript as a Supporting Information file to be published alongside your study, and cite it in the Methods section. A legend for this file should be included at the end of your manuscript.

b) If no such document exists, please make sure that the Methods section transparently describes when analyses were planned, and when/why any data-driven changes to analyses took place.

c) In either case, changes in the analytical approach -- including those made in response to peer review comments-- should be identified as such in the Methods section of the paper, with rationale.

This paper does not include data analysis and hence no prospective analytic plan was used nor were data-driven changes made. We added the following text to Methods - Disease simulation:

"All scenarios are based on hypothetical test-trace-isolate programs designed to represent a range of possible program effectiveness. Other parameter assumptions are available in Table S3."

**As noted above for the abstract - it's not clear that one of the main limitations raised by reviewers - that of adherence to isolation measures - is taken into account in the models. If not, and/or if only done so to a limited extent, we'd suggest including this in the Limitations section as the reviewers note.*

As detailed below, we added a new supplemental analysis that examines the impact of quarantine duration on onward transmission, and added clarifications on limitations related to adherence in the Methods, Results, and Discussion. The quarantine duration model feature was also added to the R package and the web application.

Comments from the reviewers:

Reviewer #1: The authors present a mathematical modeling framework aiming to evaluate the expected reductions in the reproductive number, R, from test-trace-isolate programs.

Comments:

This is a well written, important and timely article. The mathematical model is presented clearly and concisely. The framework appears to be appropriate for the requirements of this modelling exercise.

Did the authors consider 'adherence or compliance to isolation or quarantine requests' as a parameter of interest?

> As isolation and quarantine adherence are not metrics that would be readily measurable by most testing and contact tracing departments, our primary results display perfectly effective isolation and quarantine (infinite duration). We have added to the last paragraph of Methods - Mathematical framework to clarify this:
>
> "We therefore assume isolation and quarantine are perfectly effective, such that individuals do not transmit once in quarantine or isolation, though exceptions to this assumption are discussed below."
>
> Imperfect isolation and quarantine adherence could be represented in multiple forms -- delayed start, early end, or imperfect isolation from contacts. The "delayed start" is currently embedded in the \tau_D and \tau_Q parameters (time from case symptom onset to case isolation or contact quarantine).
>
> To model the "early end" scenario, we implemented a model extension which enables a tunable average quarantine duration. This new feature enables users to better capture their local guidelines on quarantine duration, or to set assumptions about imperfect quarantine in their population. This new feature was also added to ConTESSA (modifiable in the Advanced Options) and the *tti* R package. As described in the supplement (eqn. 33), this feature modifies the proportion of contacts which are considered effectively quarantined (that is, some proportion of truly infected contacts will leave quarantine before they develop symptoms and know they are infected).
>
> We also added a new Figure S4 to display the impact of quarantine duration under different scenarios, which is accompanied by the following text under Results - Adding real-world complexity (last paragraph):
>
> "Recent policy recommendations have suggested that 10-day quarantines rather than the previously-recommended 14-day quarantines may be acceptable under certain circumstances. When we modify our assumption of perfectly effective quarantine, we

find that 10-day quarantines are somewhat less effective and that this effect is more pronounced in scenarios with rapid quarantine and widespread and rapid isolation (Figure S4).”

We do not incorporate any explicit parameters to represent “imperfect isolation and quarantine” as these will not typically be known by a public health department. Those that wish to account for this kind of imperfect adherence manner can adjust the “proportion quarantined” and “proportion isolated” parameters. We have added the following text as the second paragraph in Methods - Adding real-world complexity to explain how the model parameters should be modified to account for imperfect quarantine and isolation:

“Contact quarantine may not perfectly disrupt onward transmission in the real world; average quarantine duration may be modified in the expanded model to match local guidelines, and imperfect quarantine adherence may be incorporated by tuning the proportion of contacts assumed to be *effectively* quarantined. The assumption of perfectly effective case isolation may be modified by tuning the proportion of cases assumed to be *effectively* isolated.”

We also added the following text to the Supplement (following eqn 21) to describe how tuning the overall proportion isolated and quarantined can incorporate imperfect adherence:

“In both cases, it is assumed that case isolation is perfectly effective (that is, all transmission is stopped once a case is isolated). This assumption can be relaxed by reducing the proportion of cases assumed to be isolated or quarantined by the assumed reductions in isolation effectiveness (that is, isolating 50\% of cases at 100\% effectiveness is equivalent to isolation 100\% of cases at 50\% effectiveness).”

We also added the following statement of limitations to paragraph 8 of the Discussion:

“Though non-adherence can be crudely considered by modifying the proportion isolated and proportion quarantined, our model framework does not capture the real-world imperfections where cases and contacts are unable to completely avoid contact with all other individuals.”

The model treats all cases as equal, and does not include any element of behavioural effect or community exposure. For instance, a case where someone is working from home without the need to travel and with no dependents nor contacts in the community is different to a case where someone uses public transport on a daily basis to a communal workplace, with children at school, and an active social life.
This is acknowledged to some extent, but could be expanded upon, within the limitations, which state: "This model describes a general strategy of tracing and quarantining the immediate contacts of identified cases in a community. It may not be easily extensible to settings such as

schools or workplaces. ... The model relies on a simplified version of transmission that does not account for many risk factors for SARS-CoV-2 infection or the contact structure in the population, which could lead to persistent transmission even when the population reproductive number is low."

> While the primary results use a fixed reproductive number, the stochastic version of the model does enable overdispersion in transmission, which would account for compartment-level variability in the contact structure and other factors that cause heterogeneous transmission in a population. A comparison of the deterministic and stochastic models in Figure S3 showed that these results were comparable in the mean (where the overdispersion parameter for negative binomial draws for the number of onward infections for each INFECT compartment was set to 0.1). The implementation of this model is described in the second paragraph of Methods - Disease Simulation.
>
> Nevertheless, we agree that the model estimates population-level effects and does not capture individual-level variability in behavior or test-trace-isolate effectiveness. We've expanded the limitations in paragraph 8 of the Discussion:
>
> "The model relies on a simplified version of transmission that does not account for many risk factors for SARS-CoV-2 infection or the contact structure in the population, which could lead to persistent transmission even when the population reproductive number is low (e.g., if there are clusters of connected cases that are not detected or cannot isolate completely). This may be particularly true if heterogeneities in transmission are associated with our ability to identify individuals through testing or contact tracing"

Furthermore, the authors acknowledge that "while the reproductive number is a useful representation of transmission control at the population level, it does not capture differential health burden of infections, and a program could have a higher R but better limit mortality if it effectively protects at-risk populations." These caveats for accurate interpretation and application of the model should also be stated more clearly throughout the manuscript.

> In the last paragraph of the Introduction, we now state "Here, we propose a mathematical framework for modeling the impact of test-trace-isolate strategies on onward transmission, as measured by expected reductions in an average, population-level reproductive number.", to clarify that our work is focused at a programmatic level.

Overall, this study provides valuable insight to the elements of test-trace-isolate that have greatest impact on the transmission of SARS-CoV-2 infection, and how these elements relate with each other based on the models' assumptions of the disease natural history. Instrumentally, the model has been made publicly available, ready for further research and development, as well as the exploration of various what-if scenarios.

Reviewer #2: *This is a nice piece of work describing a mathematical model of the potential impact of test, trace and isolate (TTI) on the reproduction number (R) of SARS-CoV-2. The work explores the impact of different levels of case detection, quarantine effectiveness and timeliness. It presents interesting findings on the proportion of contacts that are already quarantined, which is often used as a metric of TTI performance and yet depends strongly on the nature of the epidemic (e.g. R) and may in fact be inversely related to some measures of performance such as timeliness. The work is also supported by the provision of an online tool and comprehensive coursera course, which really increase the utility of this work. I would therefore recommend publication.*

I have just a few comments. Firstly, it would be helpful to present the separate impact of case isolation (D compartment) and quarantine of contacts (Q). Typically the latter will be smaller than the former, which could be achieved without TTI if symptomatic individuals self-isolated. This would help understand the added value of TTI over just isolation based on symptoms.

> As test-trace-isolate activities likely impact rates of self isolation based on symptoms (e.g., by increasing public awareness), and these influences may be difficult to disentangle, we decided to retain the existing primary scenarios in the manuscript. Figure 3 does allow for assessment of the separate impact of contact quarantine and case isolation under a given isolation or contact quarantine scenario (Panels A, B, D, E), including scenarios where contact tracing is extremely limited. As we note in the text, it is difficult to draw unilateral statements about whether case isolation vs contact quarantine will have greater impact, as the effects of each are highly intertwined. Further, the inter-relatedness of these metrics means that, when shown individually, there may be unusual edge case results that could be subject to mis-representation.

Secondly, it is likely that willingness to be tested and self-isolate is related to the probability of being identified as a contact and being willing to quarantine (i.e. rho and omega in the detection matric are likely to correlated at the individual level). This will limit the impact of TTI and should be mentioned as a caveat.

> We've expanded the limitations in paragraph 8 of the Discussion:
>
> "The model relies on a simplified version of transmission that does not account for many risk factors for SARS-CoV-2 infection or the contact structure in the population, which could lead to persistent transmission even when the population reproductive number is low (e.g., if there are clusters of connected cases that are not detected or cannot isolate completely). This may be particularly true if heterogeneities in transmission are associated with our ability to identify individuals through testing or contact tracing"

Finally, there is related, published work that uses a similar framework and comes to similar conclusions that ought to be mentioned (doi:10.1016/S1473-3099(20)30630-7).

Thank you for providing this reference. We have added the following reference in the paragraph 1 of the Discussion:

“Nevertheless, exceptional performance may be needed across all of these dimensions if transmission is to be controlled by test-trace-isolate alone, raising the importance of complementary control activities.”

Reviewer #3: Review of 'Maximizing and evaluating the impact of test-trace-isolate programs'.

In this manuscript, the authors develop a mathematical framework for analysing a test trace isolate (TTI) programme for COVID, aimed at helping public health authorities work out the weak spot of their programmes and improve them. There are two broad conclusions 1/ to be really effective, TTI can't have too many weak spots at all, and 2/ if the testing component is weak, fix that before looking at anything else.

The mathematical framework is based on a next generation matrix formalism, and the outcome of interest is the effective reproduction number R. The paper is accompanied by an R package, an online page, and a Coursera online course; really useful work.

I think this is a very useful paper, very well put together. Here are some comments, hope they help improve an already excellent paper.

You assume, but don't say in words, that self isolation and quarantine perfectly stop further transmission (can see in equations 5 and 6). That needs to be stated. I also wasn't too sure how to think about imperfectly effective isolation and quarantine in your framework. Please discuss (including in the Contessa website).

> As isolation and quarantine adherence are not metrics that would be readily measurable by most testing and contact tracing departments, our primary results display perfectly effective isolation and quarantine (infinite duration). We have added to the last paragraph of Methods - Mathematical framework to clarify this:
>
> “We therefore assume isolation and quarantine are perfectly effective, such that individuals do not transmit once in quarantine or isolation, though exceptions to this assumption are discussed below.“
>
> Imperfect isolation and quarantine adherence could be represented in multiple forms -- delayed start, early end, or imperfect isolation from contacts. The “delayed start” is currently embedded in the \tau_D and \tau_Q parameters (time from case symptom onset to case isolation or contact quarantine).
>
> To model the “early end” scenario, we implemented a model extension which enables a tunable average quarantine duration. This new feature enables users to better capture their local guidelines on quarantine duration, or to set assumptions about imperfect quarantine in their population. This new feature was also added to ConTESSA (modifiable in the Advanced Options) and the *tti* R package. As described in the supplement (eqn. 33), this feature modifies the proportion of contacts which are considered effectively quarantined (that is, some proportion of truly infected contacts will leave quarantine before they develop symptoms and know they are infected).

We also added a new Figure S4 to display the impact of quarantine duration under different scenarios, which is accompanied by the following text under Results - Adding real-world complexity (last paragraph):

“Recent policy recommendations have suggested that 10-day quarantines rather than the previously-recommended 14-day quarantines may be acceptable under certain circumstances. When we modify our assumption of perfectly effective quarantine, we find that 10-day quarantines are somewhat less effective and that this effect is more pronounced in scenarios with rapid quarantine and widespread and rapid isolation (Figure S4).”

We do not incorporate any explicit parameters to represent “imperfect isolation and quarantine” as these will not typically be known by a public health department. Those that wish to account for this kind of imperfect adherence manner can adjust the “proportion quarantined” and “proportion isolated” parameters. We have added the following text as the second paragraph in Methods - Adding real-world complexity to explain how the model parameters should be modified to account for imperfect quarantine and isolation:

“Contact quarantine may not perfectly disrupt onward transmission in the real world; average quarantine duration may be modified in the expanded model to match local guidelines, and imperfect quarantine adherence may be incorporated by tuning the proportion of contacts assumed to be *effectively* quarantined. The assumption of perfectly effective case isolation may be modified by tuning the proportion of cases assumed to be *effectively* isolated.”

We also added the following text to the Supplement (following eqn 21) to describe how tuning the overall proportion isolated and quarantined can incorporate imperfect adherence:

“In both cases, it is assumed that case isolation is perfectly effective (that is, all transmission is stopped once a case is isolated). This assumption can be relaxed by reducing the proportion of cases assumed to be isolated or quarantined by the assumed reductions in isolation effectiveness (that is, isolating 50\% of cases at 100\% effectiveness is equivalent to isolation 100\% of cases at 50\% effectiveness).”

We also added the following statement of limitations to paragraph 8 of the Discussion:

“Though non-adherence can be crudely considered by modifying the proportion isolated and proportion quarantined, our model framework does not capture the real-world imperfections where cases and contacts are unable to completely avoid contact with all other individuals.”

The swap from calendar time to generation time is a neat trick in epidemiology (see e.g. Pellis Math Bioscience 2008), and used very effectively here. I was nonetheless left a bit puzzled by the incorporation of delays, and given the conclusions, I would have liked to understand this better. In short, I didn't see any detailed treatment of the delays beyond equations (5) and (6), which are only half defined, since f(x) and g(x) are not defined. I would have liked to see quite a bit more detail here to be satisfied that these results are reproducible. I would also have liked to see what effects the fact that tau_D and tau_Q are themselves distributed, and to have seen more information about the functions f(x) and g(x). We have found in our simulations that the variances of incubation, generation time, and delay times, all have quite a big effect. This was pointed out in Fraser et al PNAS 2004 cited here, and seems to be relevant for COVID.

We added more details on the infectiousness distributions for index cases and quarantined contacts (functions f(x) and g(x), respectively) in the supplement (eqn. 19-21; Recursive propagation of infections) and to Table S3 and clarified the notation therein. We note that He et al., which informed our distribution of infectiousness, issued a correction to their infectiousness distribution parameters after our initial submission, and all of our figures, tables, and results have been updated to reflect this change (keeping our serial interval the same as before).

We also added the following description to the Supplement (following eqn 19).

"We derived the distribution of infectiousness (Gamma: shape = 21.13, rate = 1.59, offset = -12.27) relative to symptom onset from a previously published work \cite{He2020}, which had different estimates of the generation time (5.8 days to our baseline 6.5 days) and incubation period (5.2 days to our baseline 5.5 days) (Table \ref{tab:parameters}). We aligned their estimate for the infectiousness distribution to our generation time and incubation period assumptions, by holding the rate parameter constant and solving for the shape parameter for f(x) in the equation that follows:

generation time = E(X) + incubation period

where X is the gamma-distributed random variable representing time from primary symptom onset to secondary infection.

We used these same parameters to develop a distribution of infectiousness of secondary cases of type y, g(x), as a function of the time from their infector's time of symptom onset to contact quarantine, \tau_{Q(y)}. Once again assuming a gamma distribution, we hold the rate equal to 1.59 and offset equal to -12.27 for both f(x) and g(x) and solve for the shape of g(x) using the incubation period and shape, rate, and offset of f(x)."

We agree that variability in the isolation and quarantine delay distributions could have an important impact on the model, but these metrics are not typically collected or reported by public health departments. We opted for simple mean assumptions here to prioritize

utility for end users. We added the following sentence on this limitation to paragraph 8 in the Discussion:

"We assumed that the incubation period, generation time, and test-trace-isolate time delays were fixed at mean values, although previous work has shown that variability in these time delay distributions may affect the impact of disease control \cite{Fraser2004}."

There is a sensitivity analysis to different choices of generation time etc, but I would still nonetheless say that the baseline choice is a bit odd for COVID-19, consisting of a long generation time estimate (6.5 days, unreferenced?) compared to many publications (see e.g. Ganyani et al Eurosurveillance and many others), and effectively no asymptomatic infection. I wonder if the authors could rejig which are their baseline assumptions and which are sensitivity analyses.

The 6.5 day generation time comes from a serial interval estimate of 48 index and secondary case pairs identified in Shenzhen, China from January-February 2020 (Bi et al. doi: https://doi.org/10.1016/S1473-3099(20)30287-5). While other studies estimating the serial interval may have required decision rules when exposure data were missing or reports were censored, we preferred the estimate from this study because it included only clear case-contact pairs and there were clear dates for first exposure, last exposure, and symptom onset. Our default is also consistent with the estimate used for the distribution of infectiousness, which estimated a mean serial interval of 5.8 days (He et al. doi: https://doi.org/10.1038/s41591-020-0869-5). We added references for our model parameters in Table S3.

The reviewer is correct in stating that our primary model results make some very simplified assumptions -- they do not differentiate between asymptomatic and symptomatic transmission or between risk among household and community contacts, and R is a fixed value instead of a stochastic draw with overdispersion. Our aim with this simplification was to draw readership from public health practitioners who would be more interested in understanding big picture results rather than delving into detailed model assumptions. While specific estimates of R may change when adjusting these assumptions to something more realistic, we do not find that the overall recommendations on how to improve TTI change. Further, the conceptual diagram in Figure 1 shows only a 3 compartment model (for ease of understanding) and the primary model settings and results directly match the model depicted there. We prefer to keep our baseline assumptions as they are, but we will defer to the editor's preference on this matter.

Initially I was puzzled by the results which include focus on areas of parameter space where testing is poor, isolation is imperfect, and yet tracing and quarantine is highly effective. Yet, I can imagine this happening in practice due to these engaging very different expertise, and

different public health departments. So in some ways it shouldn't need to be said that you then need to test more and faster, but it's good to set it out, and the findings here are clear.

> Thank you for this comment. We were indeed thinking of situations where testing and contact tracing might be conducted by separate arms of a public health department, as these circumstances were most relevant to the partners with whom we were engaging in this work. To clarify this point, we include the following sentence to paragraph 1 of the Discussion:
>
> "Here, we have presented a modeling framework with which to evaluate the performance of the often independent test-and-isolate and contact tracing components of test-trace-isolate programs [...]"

Sup Table S3 does not say what value was used for the proportions of asymptomatic and symptomatic infections that are detected by surveillance, and I don't understand why. These two parameters are presumably different from each other and relevant for the results.

> These are parameters which vary throughout the multiple scenarios presented in the paper. We have added a footnote to the table to clarify which parameters are fixed and variable in the results presented, including that the proportion of asymptomatic and symptomatic individuals are equal except in Supplementary Figure S1. For simplicity, in the main text, we assume that there are no differences in detection of asymptomatic and symptomatic individuals. This assumption is explored in Fig S1.

The bottom half of Fig 2 should say quarantine instead of isolation. In Figs S1 and S2 the labels for the colours in the legend does not match the labels for the colours in the plot.

> To be consistent in our language, we use 'isolation' for any individual that is a suspected or confirmed case. In the representation in Figure 2, because the infected contact is assumed to be traced after symptom onset, we use 'isolation' in the figure. We have clarified in the figure legend:
>
> "Individuals in generation *t+1* are then *traced* and quarantined (and subsequently isolated, if the contact is a suspected or confirmed case) to reduce onward transmission from those who may be infected."
>
> In Figures S1 and S2, the labels in each plot show the percent of *all* infections isolated (Fig S1) or quarantined (Fig S2), while the lines are colored by only the proportion of asymptomatic infections isolated (Fig S1) or community contacts quarantined (Fig S2). We have added additional labels to each figure to clarify the interpretation.

I haven't tried the R package. I think someone should before publication. It sounds like the Coursera materials have already been road tested, which is great. I tried the shiny app, very slick, though I wonder if some of the parameters are a bit obscure; e.g. the difference between

being tested and being isolated. In our jurisdiction, people should isolate immediately after the test, a problem we face is that many don't.

The R package underlies the Shiny app, and thus has undergone fairly extensive testing. We do welcome continued review, though!

The Shiny application was designed for use by public health officials and decision makers who are relatively less interested in technical model details, but very familiar with programmatic testing and contact tracing metrics, including the difference between receiving a positive test and being isolated. We piloted the app with several intended users prior to its public launch and these discussions helped shape the language describing the data inputs. We encourage users of the application to take the free Coursera course, which explains all of the model parameters and how to modify the advanced options in detail. ConTESSA users may additionally find methodological details in the About page (including a link to the full model description), or to download a report which presents a table with all of the data inputs and model assumptions.
